# Geographic Variation of Female Genital Mutilation and Legal Enforcement in Sub-Saharan Africa: A Case Study of Senegal

**DOI:** 10.4269/ajtmh.14-0074

**Published:** 2015-04-01

**Authors:** Ngianga-Bakwin Kandala, Paul N. Komba

**Affiliations:** Division of Health Sciences, Warwick Medical School, University of Warwick, Coventry, United Kingdom; Populations, Evidence and Technologies Group, Warwick Evidence, Coventry, United Kingdom; Division of Epidemiology and Biostatistics, School of Public Health, University of the Witwatersrand, Johannesburg, South Africa; Wolfson College, Centre of African Studies, University of Cambridge, Cambridge, United Kingdom

## Abstract

This paper draws on household data to examine the prevalence of female genital mutilation (FGM) in Senegal and the effectiveness of the country's anti-FGM law in dealing with actual breaches and providing protection to the victims. The 2010–2011 Senegal Demographic Health Survey and Multiple Indicators Cluster Survey (SDHS-MICS) covers 14,228 women and their daughters. Logistic regression was used to investigate the geographic distribution of FGM across regions. For the enforceability of anti-FGM, desk research was used. Overall prevalence among women and daughters was 28.1% and 6.2%, respectively. Significant factors were sociodemographics, ethnicity, and region. This analysis shows both advantages and vulnerabilities of the anti-FGM law in relation to the issue of enforcement. It indicates that the law falls short of offering adequate protection to potential victims. FGM is a cultural and social norm imbedded predominantly in rural settings and as such, drives resistance to jettisoning FGM. Legislation has been one of the driving forces behind the eradication of the practice.

## Introduction

Female genital mutilation (FGM) has widely been described as female genital cutting (FGC) and refers to practices involving the partial or complete ablation of the external female sexual organs on non-medical grounds. The World Health Organization (WHO) distinguishes among four types of FGM based on the invasiveness of the intervention, and these are described elsewhere.^1^–^3^ With the benefits of modern Western medicine, women who have suffered mutilation can be treated. Some experts suggest that specialist surgeons can reverse many of the FGM consequences.^4^,^5^ However, the view in this paper is that the procedure is irreversible and that the modern medical procedures cannot replace the natural clitoris and remove the long-term psychological and physical effects caused by FGM. Even if this was the case, such treatment may not be available in Senegal and other areas where FGM is prevalent. Also, even where available, such treatment would not necessarily be attractive or requested by those who do not regard FGM to be a medical problem. According to recent estimates, between 100 and 140 million girls and women today are forced to undergo FGM or circumcision.^6^,^7^

Likewise, on the side of the law, it is suggested that no amount of legislative measures would be sufficient to completely eradicate FGM. This is because FGM, once performed, is irreversible.^3^ All that the law can do is perhaps dissuade or discourage the recurrence of the practice across present and future generations. It is in this sense that some international organizations consider the law to be one aspect of an effective campaign against FGM around the world.^8^ Among certain ethnic groups in Senegal, caregivers and families are still prepared to embrace the FGM procedure as part of accepted social norms, although it has tragic consequences (e.g., life-threatening hemorrhage, disabilities, and difficulties, such as infant death and medical complications at birth). FGM is carried out on girls at different ages ranging from infants and toddlers to teenagers. It is frequently carried out in unsterile conditions by traditional and non-medically trained practitioners.^1^,^3^,^6^,^9^–^20^ The fact that FGM is conducted in such conditions is both the result of its traditional practice, especially in Sub-Saharan Africa (SSA), and its illegality.

In other words, public health burdens of FGM include both consequences for mother or daughter mortality and ongoing morbidity concerns throughout their lifespans. As a potentially life-threatening procedure, FGM is now widely condemned as a violation of human rights and a criminal offense in many national jurisdictions.^2^

Various studies give many reasons for practicing FGM around the world. One overriding reason in Africa and elsewhere is that marriage does not come easily without the female having to prove her virginity.^20^ Other studies point to other factors, such as an initiation of girls into womanhood; traditional views on female decency, control of female sexuality or reduction of female sexual desires, enhancement of fertility, and child survival; religious requirements, especially for Muslim populations; a prerequisite for marriage in some communities, like the Fulani people of Senegal; and a means of avoiding perceived uncleanliness.^20^–^24^

This paper examines the geographic variation of FGM and the effectiveness of an anti-FGM law in Senegal. The intention is to provide information for specific and targeted intervention in that country. Existing research does not sufficiently reflect on household data to determine the degree of prevalence of such a practice. It also suggests that the anti-FGM law has largely failed to secure meaningful prosecution^25^ and that abandonment should be secured through harmonization between the law and efforts by non-government organization (NGO) initiatives to engage in educational and awareness campaigns with local communities in Senegal. The main point of this paper is to revisit some of these claims, especially one relating to the ineffectiveness of this law, and reassess the question of both the prevalence of the FGM practice and the contribution of any anti-FGM law in stamping out the FGM practices across communities. We shall use the national representative household data of Senegal to examine country geographic variation at the regional level to highlight factors associated with the practice and the role of preventive programs for policy recommendations. The choice for Senegal as a case study is based on the realization that the country represents today a limited success story in matters regarding reduction in the prevalence of FGM. The success is ascribed to combined efforts by NGOs and government bodies to build awareness among communities about the adverse human rights and health consequences of FGM and commit these communities to initiatives designed to abort the practice. This means that reduction in the FGM prevalence is not based on any idea that legislation alone is successful but different ways of enforcing such laws in dialogue with communities. It is anticipated that other countries can learn from Senegal to reduce the scale of FGM practices by looking at different enforcement mechanisms.

## Background Information about Senegal and FGM in Senegal

Before exploring how Senegal has tackled FGM, we first provide some background information about Senegal and then present a small situation analysis of the prevalence of FGM in the country. In essence, the Republic of Senegal is located on the coast of western Africa with a population of about 13 million, of which 94% are Muslims, 4% are Christians, and 2% belong to traditional religions. There are about 20 different ethnic groups distributed across 14 provinces. The largest ethnic groups are the Wolof, the Fulbe and Toucouleur, the Serer, the Fulani, the Diola, and various Mandingo groups. Women are disadvantaged in both economic and societal terms as a result of their status and the prevailing sociocultural norms. The literacy rate (defined as the percentage of persons aged 15 years old and over who can read and write) is 33% for women and 52% for men. The percentage of women aged 20–24 years old who were married before the age of 18 years old is 39%. Senegal has a population growth rate of about 2.5% per year and a total fertility rate of 4.6 children born per woman.^26^ With a gross national product (GNP) per capita of US$1,900 (2012 estimation), around two-thirds of the population live on less than US$1 a day, and Senegal is among the 30 least economically developed countries in the world, ranking 154 of 186 in 2012 in terms of the human development index (HDI).^26^ Life expectancy at birth is 63 years, and the national poverty rate is 46.7%.^26^ Senegal is a country with a huge gender inequality index (youth population [millions; 15–24 years old]: 65%; female youth, 56.2%; male youth, 74.2% [2009]; under 5 years old mortality rates [per 1,000 live births]: 55.16). Maternal mortality ratio is estimated at 370 deaths of women per 100,000 live births.^26^,^27^

Most women who are cut in Senegal undergo excision (type II according to the WHO classification). This involves the partial or total removal of the clitoris and the labia minora. About 12% of women stated that they had been subjected to infibulation (type III according to the WHO classification; i.e., narrowing of the vagina with [partial] removal of the labia minora and/or majora and/or the clitoris).^1^,^3^ Almost all interventions are performed by traditional circumcisers. Only 1.3% of girls had been cut by medically trained staff. The age at which girls are cut did not vary much between the generation of mothers and the generation of daughters: almost three-quarters of women subjected to FGM in each age group had been cut before their fifth birthday. The most important reasons given for FGM were the social standing that it confers and the need to preserve girls' virginity followed by the belief that FGM is a religious duty. Of women who have been cut, 53% believe that the practice should be retained; of men, 12% believe that the practice should be retained.^3^

It is against this background that the Committee on the Elimination of All Forms of Discrimination against Women issued its General Recommendation on Female Circumcision (General Recommendation No. 14), which calls on states to take appropriate and effective measures with a view to eradicating the practice and requests them to provide information about measures being taken to eliminate FGM in their reports to the Committee.^2^,^28^ Prominent among the harms to human rights associated with FGC is the fact that those undergoing the practice in Senegal are almost entirely minors (very young children or infants at that) and incapable of meaningfully consenting to the procedure.^25^ In this context, parliamentarians from throughout Africa met in Dakar on May 3 and 4, 2010 to push for a continent-wide ban on FGM and called on the United Nations (UN) to pass a General Assembly resolution appealing for a global FGM ban to prevent violation of human rights. Members of parliaments from African nations also exchanged lessons learned and actions taken to achieve the ban and resolution. Some 17 African states have banned FGM, among them Burkina Faso, Togo, Senegal, and Uganda. Since November 26, 2012, a resolution against FGM in the UN General Assembly's human rights committee has been adopted, and it is a major boost to civil society organizations fighting for an end to the practice. This UN resolution places FGM in a human rights framework and calls for a holistic approach, stressing as it does the importance of empowerment of women, promotion and protection of sexual and reproductive health, and breaking the cycle of discrimination and violence.^24^ The rationale for a legal ban is that FGM differs from other forms of abuse of rights in that perpetrators also believe to act in the best interest of their victims. Thus, any legal ban is subject to challenges and controversy in communities where it is to be enforced.^29^

## Materials and Methods

### Materials.

The Demographic and Health Survey (DHS), funded by the US Agency for International Development (USAID), is a well-established source of reliable population-level data with a substantial focus on health. The objectives, organization, sample design, and questionnaires used in the DHS surveys are described elsewhere.^30^

The 2010–2011 Senegal Demographic Health Survey and Multiple Cluster Survey (SDHS-MICS) is a nationally representative cross-sectional survey (multistage stratified random sampling of households) of women of reproductive age (15–49 years old). The resulting sample was representative of the underlying populations of the different regions of Senegal. It was carried out between October of 2010 and April of 2011. Nationally representative samples of 15,688 women between 15 and 49 years old in all selected households and 4,929 men between 15 and 59 years old in one-third of selected households were interviewed. Response rates were over 93% for individual women and over 87% at the household level, and informed consent was obtained from participants. The study protocol conforms to the ethical guidelines of the 1975 Declaration of Helsinki as reflected in *a priori* approval by the institution's human research committee. Ethical approval was granted by the Ethics Committee of the National Statistical Office of Senegal.

Data collected were representative at the national level, the urban/rural level, and the level of each administrative region when tested using a number of sociodemographic and household indicators. There were few participants with missing data for FGM and other covariates; thus, data analysis on FGM was based on 14,228 women with a complete set of data.^30^

The 2010–2011 SDHS-MICS collected an optional additional series of questions about FGM. The history of the development of FGM questionnaire is discussed elsewhere.^30^ The questions are designed to generate information on prevalence rates and types of FGM for the women themselves and their daughters. Respondents' attitudes toward FGM are also collected. Since 2000, United Nations Children's Fund (UNICEF) MICS has used a similar module to collect information on FGM in selected countries. Both the DHS and the MICS provide FGM prevalence data. Female respondents are asked if they have ever heard of FGM; then, those who have heard of the practice are asked about their own experience of it. The responses to these questions are used to calculate national prevalence rates of FGM.^30^–^32^

Experts generally assume that women respond truthfully when asked about their own experience. If bias exists in some of the responses, it has not been documented. It is hypothetically possible that some women may not admit to having undergone FGM in countries where the practice has been forbidden, but no solid evidence of this has been found.

The module on FGM included questions on whether the woman herself had undergone FGM and if she had daughters, whether they had also undergone the practice (Table 1) or whether she intended that they should.

We use the binary outcome whether a woman or dependent daughter has undergone FGM, and for the daughters' analysis, the unit of analysis was circumcised daughters from any women because of interpretability reasons, because with the binary outcome, one can estimate the likelihood of FGM in a given region of Senegal, while accounting for a number of potential covariates.

**Figure 1.**
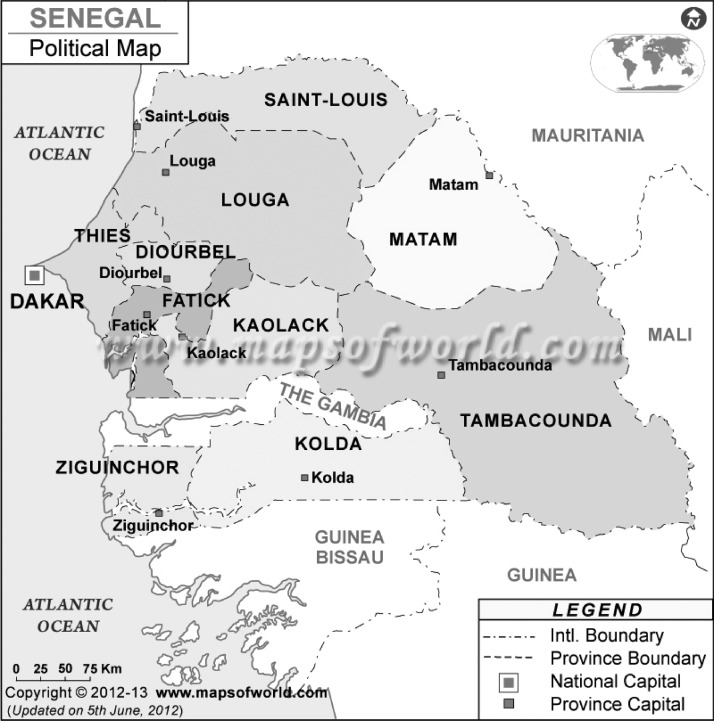
Political map of Senegal.

The main exposure variable investigated was the respondent's geographic location (i.e., the region of residence at the time of the survey) (Figure 1) in addition to various individual-level control variables, such as sociodemographics known to be associated with FGM. The respondent and her partner's age at the time of the survey were also included as an indicator of the birth cohort of the women. Other sociodemographic covariates were religion (Catholic versus other Christian, Islam, traditionalist, and other), wealth index (poorest versus poorer, middle, richer, and richest), and education of the respondent and partner (no education versus primary, secondary, and higher education). Finally, environmental factors included place (locality) of residence (rural versus urban) and region of residence of the women, including her ethnicity.

### Methods.

To account for geographic variation in the prevalence of FGM at the regional level in Senegal, we used a logistic regression model to investigate the geographic distribution of FGM across the 14 regions while accounting for individual-level risk factors. The response variable was defined as *y*_i_ = 1 if the woman/daughter was circumcised and *y*_i_ = 0 if the woman/daughter was not circumcised. The standard measure of effect was the odds ratio (OR) and 95% confidence interval (95% CI).

The analysis was carried out using the STATA 12 software package (Stata Corp, College Station, TX). The statistical significance of associations between potential risk factors and the prevalence of FGM was explored with χ^2^ and Mann–Whitney *U* tests as appropriate. Adjusted marginal ORs of FGM risk across regions were obtained from standard logistic regression models, with Diourbel used as the reference category because of its lowest crude FGM prevalence (Table 2).

## Results

Baseline characteristics of the study population are displayed in Table 1 (weighted data) for the overall sample and their daughters (*N* = 14,228) and in Table 2 (unweighted data) with participants split within the two categories of circumcised women versus not circumcised, with circumcised status of daughters listed in Table 3 (*N* = 14,228). The bivariate results show that, overall, 5,689 (i.e., 39.98%) of women had undergone FGM; 1,340 (9.4%) women reported that they had daughters who had undergone FGM, and 3,330 (23.4%) women reported that they intended the practice of FGM continue. Overall, mean age of participants was 27.9 (0.09) years, and for their partners, the mean age was 43.5 (0.17) years. The percentage of participants with no education was high (57.9%), and it was 75.4% among their partners; 49.3% of participants were living in an urban area, 95.4% were Muslim, 16.5% were in the poorest quintile of the wealth index, and 38.7% were from the Wolof ethnic group. Prevalence values of FGM among respondent women and daughters were 28.1% and 6.2%, respectively, with variation across provinces and ethnic groups ranging from 0.7% in Diourbel to 92.0% in Kedougou and from 2% among the Wolof to 89.6% among the Mandingue ethnic group for respondent women. For daughters, the prevalence of FGM varies from 0.3% in Thies to 26.9% in Sedhiou and from 0.2% among the Serer/Wolof to 22.1% among the Mandingue ethnic group.

On average, circumcised women were older than their non-circumcised counterparts and their partners, more likely to have a lower education and have non-educated partners, more likely to be living in rural areas, and more likely to be Muslim. In addition, circumcised women were more likely to be in the poorest quintile of the wealth index, from the Mandingue ethnic group, and living in Kedougou (Table 2). For the daughters, on average, circumcised daughters were from older women and partners than their non-circumcised counterparts, more likely to come from non-educated parents, more likely to be living in rural areas, and more likely to be Muslim. In addition, circumcised daughters were more likely to be in the poorest quintile of the wealth index, from the Mandingue ethnic group, and living in Kedougou (Table 2).

Table 3 displays both ORs of FGM among women and daughters across the selected study characteristics. Results of both multivariate logistic regression analyses of women and daughters do not support the role of modernization (women's wealth and education) and religion as factors associated with FGM. However, our results support the idea that FGM is a cultural norm, and living in a rural setting is a risk factor for the likelihood of being circumcised for both women and their daughters.

Specifically, women from the Mandingue ethnic group (OR = 96.06; 95% CI = 69.0, 133.7) and the Soninke ethnic group (OR = 72.0; 95% CI = 49.3, 105.1) followed by the Diola (OR = 40.4 ; 95% CI = 29.1, 56.0) and Poular/Fulani ethnic groups (OR = 37.6; 95% CI = 30.2, 46.8) from rural settings (OR = 1.18; 95% CI = 1.01, 1.38) were consistently associated with higher odds of being circumcised. Also, daughters from the Poular/Fulani ethnic group (OR = 6.79; 95% CI = 4.93, 9.35) from the rural settings (OR = 1.24; 95% CI = 1.04, 1.48) were consistently associated with higher odds of being circumcised than their counterparts of the Madingue and Soninke ethnic groups. The associations of women and partner education, wealth index, and religion with FGM risk were not statistically significant. Moreover, there were linear associations between women's age and FGM status: the risk of undergoing FGM increased with age. In other words, older women from the age group 36–49 years (OR = 1.37; 95% CI = 1.16, 1.62) and age group 26–35 years (OR = 1.18; 95% CI = 1.02, 1.37) were more likely to be circumcised compared with their counterparts in the younger age group less than 25 years old. Also, daughters from older women (age group = 36–49 years; OR = 2.61; 95% CI = 2.16, 3.16 and age group = 26–35 years; OR = 3.07; 95% CI = 2.60, 3.61) were more likely to be circumcised compared with daughters from younger women (age group less than 25 years old) (Table 4).

With regard to FGM status, in the regression analyses, there was a striking variation in FGM risk across regions for both women and their daughters, with the highest risk being in Kedougou region (OR = 162.0; 95% CI = 98.0, 269.7) followed by Tambacounda (OR = 84.0; 95% CI = 56.9, 124.2), Sedhiou (OR = 64.3; 95% CI = 42.9, 96.5), and Kolda (OR = 62.5; 95% CI = 42.0, 91.4) and the lowest risk being in Louga (OR = 0.78; 95% CI = 0.52, 1.18) and Diourbel for women respondents. For daughters, the highest risk was in Sedhiou region (OR = 3.5; 95% CI = 2.38, 5.33), Kolda (OR = 2.90; 95% CI = 1.97, 4.26), and Tambacounda (OR = 2.84; 95% CI = 1.93, 4.18) and the lowest risk was in Kaolack region (OR = 0.08; 95% CI = 0.03, 0.22) and Thies (OR = 0.11; 95% CI = 0.04, 0.31).

The above results for covariate-adjusted province variation of FGM status show a clear pattern of regions with higher risk of FGM (mostly the southeastern regions of Kolda, Sedhiou, and Tambacounda, including the eastern region of Kedougou, which was associated with a higher prevalence of FGM, whereas provinces in the west and north were associated with a lower FGM prevalence). These geographic patterns confirm the observed and model findings shown in Tables 2–4 for both women and daughters.

Even after multiple adjustments of the urban environment and other risk factors, findings for both women and daughters confirm the fact FGM is lower in the largest urban province Dakar.

## Discussion

This combined epidemiological and legal study offers a unique opportunity to examine the geographic variation of the FGM prevalence and the effectiveness of the law in Senegal. We used the most recent data from the 2010–2011 SDHS-MICS, a large nationwide sample of women and their children across the 14 regions of Senegal. Then, we considered the effectiveness of the legal responses to the issue of FGM prevalence in that country.

The overall prevalence of FGM in Senegal was 28.1% using the most recent survey, the 2010–2011 SDHS-MICS. However, according to the DHS conducted in 2005, 28% of women aged between 15 and 49 years old in Senegal have been subjected to FGM. Comparing the previous survey with the recent survey, it indicates a zero change in prevalence in the 5 years separating the surveys. However, there were large geographic variations within the country by region. For instance, Kolda region changed from a prevalence of 94% in 2005 to a prevalence of 86%, an 8% decrease during the 5-year period. There were also other regions with similar decreases in percentages, such as Tambacounda. However, the prevalence of FGM was still high, even after Senegal signed on to the Maputo Protocol in 2006. According to the 2010 DHS data, in urban areas, the prevalence was 35.6%, and rural residents reported a prevalence of 43.1%.

In this study, we investigated social, demographic, and economic factors associated with FGM in Senegal. In summary, living in a particular region in the south of the country (Kedougou, Sedhou, and Kolda), living in a rural area, coming from a particular ethnicity (Madingue, Soninge, and Poular), or being a Muslim were all associated with a higher likelihood of undergoing FGM for women and their daughters. Age of the respondent, education, and household socioeconomic status were no longer associated with the likelihood of FGM after multiple adjustments of other factors.

Returning to the relationship between women's own experience of FGM and that of their daughters, we found that the difference between the percentage of younger and older women whose daughters had undergone FGM was much greater than the difference between the percentages of respondents who had undergone FGM themselves. This could represent an important change in cultural norms with decreased use of FGM, whereby younger women are less willing to have their daughters undergo FGM. This offers possible hope that the practice may be reducing over time. However, this finding could also represent a cohort effect—younger women have younger children who are not yet at risk. We found a large difference in Senegal in the risk of FGM between regions and ethnicity, with some regions and ethnic groups having a higher risk of FGM. It is worth investigating these findings further, because spatial variation and ethnicity may be indicative of an association of place of residence with FGM practice as a proxy for social norms.

## Legal Enforcement and Extraterritoriality Issues

One response to the prevalence of FGM has been to enact anti-FGM legislation. In this respect, numerous provisions exist to combat the practice of FGM. These provisions consist of international human rights standards, which Senegal has incorporated into its domestic system as well as the Senegalese 1999 anti-FGM law. The commitment to international human right standards was shown through the ratification of important treaties, such as the Convention on the Elimination of All Forms of Discrimination against Women (CEDAW), the UN Convention on the Rights of the Child (CRC), the African Charter on the Rights and Welfare of the Child, and the Maputo Protocol (to the African Charter on Human and Peoples' Rights) on the Rights of Women in Africa.

At the domestic level, the Senegalese Government adopted Article 299 of the Penal Code, which imposes a maximum penalty of 5 years imprisonment for performing FGM. Subsequently, the Ministry of Family Affairs produced and adopted Action Plan 2000–2005, according to which FGM is to be eradicated in Senegal by 2015. The main objectives were to improve networking and coordination among actors involved in efforts to combat the practice, explaining the legal framework to them and integrating the issue into formal and non-formal education. An evaluation of the Action Plan conducted in 2008 notes that, of 5,000 or so villages previously practicing FGM, a total of 3,300 had forsworn the practice by 2008 in public declarations. There are, however, still areas in which the practice is still strongly defended. It is also important to ensure the sustainability of what has been achieved. The Second National Action Plan 2010–2015, which was adopted in February of 2010, is to step-up action against FGM. The objective remains the complete eradication of the practice by 2015.^26^,^34^

Returning now to 1999 Senegalese Law no. 99–5, it should be stressed that FGM was legally called a violation of human rights, and such a law superseded social norms and the relevant provisions of the criminal code. It made carrying out FGM on a child or woman against the law and carried a maximum penalty of 5 years and/or a fine. A spokesperson for the human rights group The African Assembly for the Defense of Human Rights (RADDHO) pointed out that, although adopting the law was an important step forward, only its enforcement would ensure that women derive the most benefit from it. One way in which enforcement is achieved is through prosecution and deterrence.

However, despite this law and indeed, human right standards, FGM cases have rarely been brought before a court of law. Only a few notable exceptions exist. For example, since the law went into effect, only a limited number of arrests were made. In January 1999 the law has seen only two arrests but no convictions. In July of 1999, the public prosecutor in Tambacounda ordered the arrest of the grandmother and mother of a 5-year-old girl after a complaint filed by the girl's father alleged that the two women had ordered FGM performed on his daughter. The practitioner was also charged. After emotional public outcry in the region, however, the cases were not pursued, and no convictions resulted. The press has suggested that the passage of the law has driven the practice underground.^27^ In July of 1999, Mrs. X was reportedly convicted for allowing the excision of her young daughter. In November of 2001, three people aged 55 to 75 years old were arrested in the Velingana province for breaking the 1999 law but subsequently pardoned in the same year. Records of arrest and prosecution, however, remain patchy, and there has been no way to obtain overall nationwide figures of convictions for offenses under the law. It is, however, the case that prosecution fails, because like any crime, FGM is performed in secret and apparently not talked about in public. There are metaphors used among females who have had FGM performed.

Moreover, FGM is accepted as common as male excision, which leaves disability in many cases. Also, prosecuting authorities are the products of their communities and cultures and may not feel it a matter of public interest to expend resources to prosecute a practice that had stood the test of time and been handed down from generation to generation. Successful attempts to enforce the law depend on gathering evidence to make a positive prosecution case against the perpetrator of FGM. However, obtaining such evidence can prove exceedingly difficult. Additionally, as stated before, there is some reluctance for prosecuting officials to take on the case where the public interest test is not satisfied in enforcing the law. Such evidence might come from several potential sources. One is from health professionals who may have come into contact with these children, and the law enforcement officials look to them to blow the whistle on those suspected of having committed FGM. Another source is the victims themselves, who may have suffered as a result of the practice. The difficulty with the first source of evidence is that health professionals might be breaking their duty of confidentiality to patients. The duty to disclose information would, in that situation, conflict with the duty of confidentiality. It might be suggested that FGM is a criminal practice after the 1999 law, and therefore, confidentiality should be waived to give way to the legal duty to disclose.

Likewise, some may consider the failure to disclose as amounting to conspiracy to a crime. However, that argument is tenuous, because it would be nonsensical to call someone an accomplice when the crime was committed without their knowledge or participation. Moreover, before any charge can be brought against those who fail to report, evidence of their knowledge of FGM commission may prove difficult to establish. It is also questionable how the police can possibly collect such evidence when health professionals refuse to record FGM information or do not feel that they ought to ask the FGM question for fear of discriminating or being prejudiced against other people's culture.^35^

Apart from the paucity of prosecution cases, the 1999 law has only a limited extraterritorial effect, consistent with the French tradition on which Senegal law is based. Such law does not clearly state what happens to cases where believers in FGM cross Senegalese borders to have their daughters mutilated. In other words, the current uncertainty in Senegalese law can be illustrated as follows. A woman living in the southern regions of Senegal (e.g., Ziguinchor, Kolda, and Tambacounda) can easily cross the border into Guinea and have her daughter or herself mutilated and return safely back to Senegal. There is nothing that anyone who attempts to prosecute the perpetrator in Guinea or the person who sought his/her assistance can do when FGM is not illegal in Guinea.

The 2007 legislative reforms incorporate traditional international crimes (e.g., genocide and crime against humanity) into the Senegalese Criminal Code (UN, 2011, Convention Contre la torture et autres peines ou traitements cruels, inhumains ou degradants). The extraterritorial jurisdiction has so far been tested over the Hissene Habre saga.^36^

In principle, the notion that the state should apply justice only to offenses committed within its territorial borders can be qualified by principles, such as passive personality, where the Senegalese court would look at the nationality of the FGM victim as the basis for taking jurisdiction.^37^ The reflection of the passive personality principle would be a reformed Senegalese law stating that those taking children abroad to be mutilated will face prosecution on return to Senegal.^1^

Although the principle of passive personality applies in relation to FGM, that alone would not necessarily lead to a meaningful prosecution. This is because the issue will arise as to how to bring the perpetrator leaving abroad into Senegalese jurisdiction. Some jurisdictions have controversially resorted to kidnapping as one possible approach to such a move (see the case of Machain versus USA, 1992 S. Ct 2188). Other jurisdictions are unlikely to use this method (see the case of R v Horsferry Magistrates, 1994 1 AC 42).

One viable approach is to have recourse to extradition laws as stated in Senegalese law nos. 71–77 of December 28, 1971. However, Article 5 of this law suggests that extradition will be allowed only where the person is of Senegalese nationality and the offense has been committed on Senegalese soil (see Belgium v Senegal, General List No. 144, Judgment).^38^

Another way could be to ensure that an anti-FGM treaty is signed between Senegal and its immediate neighbors, such as the Gambia, Guinea, and Mauritania, or harmonize anti-FGM legislation at regional or African levels.

Another limitation of the current law in Senegal is that it seeks to provide legal redress for those females who have already suffered FGM and does not offer sufficient protection to females yet to be mutilated. As stated above on the point of extraterritoriality of the 1999 anti-FGM law, there is little the law would do to save Senegalese women and girls from undergoing FGM in neighboring Mauritania, Guinea, or the Gambia.

As with the laws in many countries in the region, the Senegalese anti-FGM law is more effective in terms of facilitating prosecutions of those who violate the law rather than protecting women and girls who have yet to undergo FGM from the practice. There are some efforts from the government on how to prevent FGM and provide protection for those who have not yet been cut. For policy strategies to prevent FGM, it is, instead, multilateral agencies, such as UNICEF, in conjunction with the WHO, German Federal Ministry for Economic Cooperation and Development, and NGOs, such as the United States-based Population Reference Bureau (PRB), that have played a crucial role in advocating the end of FGM in the region. This is also true for countries such as Senegal, where NGOs,^41^ such as TOSTAN, are using community-led approaches that have been proven to be effective in eradicating the practice.^2^

Despite the shortcomings of the legislation identified above (i.e., paucity of prosecution of cases and lack of sufficient protection to potential victims), it must be noted that the 1999 law and human right norms present many advantages. The first is that no national group or NGO would have been engaged in the eradication campaign unless it believed that their actions were legally justified under the Senegal law. Also, the law stands as a formal framework, which confers on public officials the power to ensure legal protection of women. Although the law is not always enforced, its existence may send deterrent signals to the perpetrators who might fear criminal prosecution. In this respect, the adoption of the 1999 law is regarded as critical to effect change of social attitudes toward FGM as a social norm.

What Senegal has achieved so far suggests that FGM can be significantly reduced in the regions identified in this paper. However, the law can only contribute to efforts to stamp out the practice if there are sufficient resources to accelerate the campaigns and secure a social contract between campaigners and targeted populations (for similar arguments, see Mackie^39^). The achievements of Senegal can be accounted for by the responses brought by various national and international NGOs,^41^,^42^ such as TOSTAN (meaning “breakthrough”, as in the hatching of an egg, in the west African language of Wolof), and civil society's positive responses to the anti-FGM standards. These NGOs' campaigns bring the knowledge of the law to the people and commit local communities to abandonment plans. Those who subscribed to the plan and then are found to practice FGM can be fined by village chiefs who act as legal enforcers at the grassroots level. TOSTAN, for example, ensures that education programs are disseminated to encourage voluntary renunciation, although education spreads fear of what might happen on the legal and healthcare fronts if FGM is practiced. The core of these education programs is to stimulate social change through non-formal education. The various modules of its Community Empowerment Program tackle FGM as both a health issue and a human rights issue.

Mostly, the education provided within the framework of the program leads to a public declaration condemning FGM. Such a declaration is deemed to be an expression of intended social change. Since 1999, GTZ (German Agency for Technical Cooperation; GTZ as of January 1, 2011)^40^ has been implementing the project Ending Female Genital Mutilation on behalf of the German Federal Ministry for Economic Cooperation and Development (BMZ). In Senegal, the GTZ's FGM project supported various activities, including TOSTAN's projects to overcome FGM in the Kolda region in the south of the country over a period of several years. After 2002, efforts focused on advising the GTZ FANKANTA (A German--Senegalese project to fight against FGM) project, which was attached to the Senegalese Ministry of Health. This project supported family planning and HIV education in various regions of the country. In Kolda region, the issue of FGM was incorporated into the project work because of the high prevalence level there until FANKANTA was replaced by an integrated Casamance-wide program in 2005.

The FGM activities that were part of the FANKANTA program aimed above all to raise the level of acceptance of the legal ban on FGM with the help of education and sensitization. In view of the difficult political situation in the Casamance, it aimed to ensure that the local population did not see the ban as a central government meddling in their affairs but something that is well-founded and rational. Thanks to this approach, FANKATA found many supporters and advocates among not only local NGOs and action groups but also, religious and social leaders who were influential within their communities. In cooperation with the project, they developed various educational materials in local languages which were used widely within the scope of numerous special events. They also made education activities possible after the project *per se* had been completed (GTZ 2011 Project: Ending Female Genital Mutilation).

This involvement of multifarious stakeholders suggests that the solution to banning FGM cannot simply be strict legal enforcement. The Senegal model can be regarded as the best exemplar for the rest of the world for dealing with FGM. The model seeks to educate and protect women by providing communities, whose members are likely to be subject to FGM, with information and knowledge about their commitment to social change. In other words, whereas in most cases, the persons convicted under the law are liable for fines and imprisonment, enforcement of the law is not satisfactory, and many believe this poor enforcement is the result of low fines, short duration of imprisonment, and sympathy of law enforcement agents because of the cultural nature of FGM. Involvement of all persons in the implementation process is equally necessary. When the practice is abandoned, communities effectively enforce new legislation and should give effect to policy strategies.

Although there is scant literature on the available policy strategies in Senegal, multilateral agencies, such as UNICEF and the WHO, have played a crucial role in advocating the end of FGM. These efforts have not yielded the desired effects, but the Community Empowerment Program in Senegal has been effective in preventing the practice. However, other obstacles remain, which lead to the conclusion that FGM cannot be completely rooted out like any crime that can be eliminated within society. These obstacles must, however, be addressed and include resistance to legal change by the traditional healers, passivity of some public officials, lack of sufficient resources at the state level to sensitize the population, and the lack of knowledge of the law banning FGM. One way to overcome lack of knowledge is to translate the law into local vernaculars and involve young men and women in the campaigns. Such campaigns have to also involve the police in their preventative rather than protective role.

## Figures and Tables

**Table 1:** Baseline characteristics of the study population (women respondents; SDHS, 2010–2011)

Variable	Women (*N* = 14,228)	Daughters (*N* = 14,228)
Mean age* (SD) for respondent	27.9 (0.09)	27.8 (9.2)
Mean age* (SD) for partner	43.5 (0.17)	43.7 (14.2)
Education respondent		
No education	57.9	62.2
Primary education	21.8	20.0
Secondary education	18.3	16.9
Higher education	2.1	0.9
Education partner		
No education	75.4	79.3
Primary education	11.9	10.4
Secondary education	9.2	7.8
Higher education	3.5	2.6
Place of residence		
Urban	49.3	39.5
Rural	50.7	60.5
Religion		
Muslim	95.4	95.4
Christian	4.2	4.0
Animist/other	0.4	0.6
Wealth index		
Poorest	16.5	23.7
Poorer	17.9	22.8
Middle	19.9	22.7
Richer	22.3	17.4
Richest	23.5	13.4
Respondent circumcised†		
Yes	28.1	28.1
No	71.9	71.9
Daughter circumcised†		
Yes	9.4	9.4
No	90.6	90.6
Ethnicity		
Wolof	38.7	33.0
Poular/Fulani	26.5	31.5
Serer	15.0	12.6
Mandingue	4.2	6.6
Diola	4.0	4.8
Soninke	2.3	2.3
Other	9.3	9.2
State of residence		
Dakar	26.0	8.7
Ziguinchor	3.7	6.0
Diourbel	11.8	9.1
Saint-Louis	6.7	6.9
Tambacounda	4.6	7.2
Kaolack	7.5	8.9
Thies	12.9	8.4
Louga	7.2	8.1
Fatick	4.6	6.7
Kolda	4.1	6.9
Matam	3.8	6.6
Kaffrine	3.7	6.6
Kedougou	0.7	3.2
Sedhiou	2.9	6.7

* Age ranges from 15 to 49 years of age.

† Data are expressed as means (SEMs) or percentages using the population weight.

**Table 2:** Baseline characteristics of the study population by women's FGM status (SDHS 2010)

Variable	Circumcised (*N* = 5,689)	Not circumcised (*N* = 8,539)	*P* value*
Mean age* (SD) for respondent	28.2 (9.3)	27.9 (9.2)	0.08
Mean age* (SD) for partner	42.7 (15.1)	43.9 (14.3)	0.46
Education respondent			< 0.001
No education	3,727 (42.4)	5,067 (57.6)	
Primary education	1,108 (38.9)	1,741 (61.1)	
Secondary education	833 (34.0)	1,614 (66.0)	
Higher education	21 (15.2)	117 (84.8)	
Education partner			0.005
No education	3,315 (43.1)	4,380 (56.9)	
Primary education	486 (46.7)	554 (53.3)	
Secondary education	337 (42.7)	452 (57.3)	
Higher education	93 (34.8)	174 (65.2)	
Place of residence			< 0.001
Urban	2,081 (35.6)	3,773 (64.5)	
Rural	3,608 (43.1)	4,766 (56.9)	
Religion			< 0.001
Muslim	5,578 (40.9)	8,064 (59.1)	
Christian	84 (16.5)	426 (83.5)	
Animist/other	27 (35.5)	49 (64.5)	
Wealth Index			< 0.001
Poorest	1,850 (55.9)	1,459 (44.1)	
Poorer	1409 (45.5)	1,691 (54.5)	
Middle	1,356 (41.7)	1,895 (58.3)	
Richer	746 (29.0)	1,829 (71.0)	
Richest	328 (16.5)	1,665 (83.5)	
Ethnicity			< 0.001
Wolof	89 (2.0)	4,464 (98.0)	
Poular/Fulani	3,243 (69.4)	1,432 (30.6)	
Serer	67 (4.0)	1,586 (96.0)	
Mandingue	909 (89.6)	105 (10.4)	
Diola	392 (59.5)	267 (40.5)	
Soninke	270 (76.3)	84 (23.7)	
Other	719 (54.5)	601 (45.5)	
State of residence			< 0.001
Dakar	266 (20.4)	1,036 (79.6)	
Ziguinchor	529 (61.8)	327 (38.2)	
Diourbel	9 (0.7)	1,284 (99.3)	
Saint-Louis	438 (45.0)	535 (55.0)	
Tambacounda	953 (87.0)	142 (13.0)	
Kaolack	86 (8.0)	994 (92.0)	
Thies	47 (3.8)	1174 (96.2)	
Louga	59 (5.5)	1,008 (94.5)	
Fatick	84 (10.1)	748 (89.9)	
Kolda	898 (85.9)	147 (14.1)	
Matam	869 (86.9)	131 (13.1)	
Kaffrine	113 (11.7)	857 (88.3)	
Kedougou	447 (92.0)	39 (8.0)	
Sedhiou	891 (88.4)	117 (11.6)	

Data are expressed as means (SDs) or percentages.

* *P* values for comparison between circumcised and not circumcised subjects.

**Table 3:** Baseline characteristics of the study population by daughters' FGM status (SDHS 2010)

Variable	Circumcised (*N* = 1,340)	Not circumcised (*N* = 12,888)	*P* value*
Mean age* (SD) for daughters	31.4 (7.6)	27.7 (9.3)	< 0.001
Mean age* (SD) for partner	45.2 (13.7)	43.6 (14.2)	< 0.001
Education daughters			< 0.001
No education	1,123 (12.8)	7,671 (87.2)	
Primary education	181 (6.3)	2,668 (93.7)	
Secondary education	36 (1.5)	2,411 (98.5)	
Higher education	0 (0.0)	138 (100.0)	
Education partner			< 0.001
No education	1,056 (13.7)	6,639 (86.3)	
Primary education	121 (11.6)	919 (88.4)	
Secondary education	66 (8.4)	723 (91.6)	
Higher education	13 (4.9)	254 (95.1)	
Place of residence			< 0.001
Urban	303 (5.2)	5,551 (94.8)	
Rural	1,037 (12.4)	7,337 (87.6)	
Religion			< 0.001
Muslim	1,320 (9.7)	12,322 (90.3)	
Christian	12 (2.4)	498 (97.6)	
Animist/other	8 (10.5)	68 (89.5)	
Wealth index			< 0.001
Poorest	548 (16.6)	2,761 (83.4)	
Poorer	368 (11.9)	2,732 (88.1)	
Middle	297 (9.1)	2,954 (90.9)	
Richer	101 (3.9)	2,474 (96.1)	
Richest	26 (1.3)	1,967 (98.7)	
Ethnicity			< 0.001
Wolof	15 (0.3)	4,538 (99.7)	
Poular/Fulani	852 (18.2)	3,823 (81.8)	
Serer	4 (0.2)	1,649 (99.8)	
Mandingue	224 (22.1)	790 (77.9)	
Diola	61 (9.3)	598 (90.7)	
Soninke	42 (11.9)	312 (88.1)	
Other	142 (10.8)	1,178 (89.2)	
State of residence			< 0.001
Dakar	28 (2.2)	1,274 (97.8)	
Ziguinchor	91 (10.6)	765 (89.4)	
Diourbel	2 (0.2)	1,291 (99.8)	
Saint-Louis	114 (11.7)	859 (88.3)	
Tambacounda	261 (23.8)	834 (76.2)	
Kaolack	4 (0.4)	1,076 (99.6)	
Thies	4 (0.3)	1,217 (99.7)	
Louga	25 (2.3)	1,042 (97.7)	
Fatick	5 (0.6)	827 (99.4)	
Kolda	251 (24.0)	794 (76.0)	
Matam	225 (22.5)	775 (77.5)	
Kaffrine	11 (1.1)	959 (98.9)	
Kedougou	48 (9.9)	438 (90.1)	
Sedhiou	271 (26.9)	737 (73.1)	

Data are expressed as means (SDs) or percentages.

* *P* values for comparison between circumcised and not circumcised subjects.

**Table 4:** Marginal ORs of women and daughters across selected covariates (SDHS, 2010)

Variable	Women marginal OR (95% CI)*	Daughter marginal OR (95% CI)†
Age groups of respondent (years)		
≤ 25	1.00	1.00
26–35	1.18 (1.02–1.37)	3.07 (2.60–3.61)
36–49	1.37 (1.16–1.62)	2.61 (2.16–3.16)
Age groups of partner (years)		
≤ 30	1.00	1.00
31–40	0.75 (0.60–0.94)	0.94 (0.75–1.17)
41+	0.68 (0.55–0.83)	0.56 (0.45–0.70)
Education respondent		
No education	1.00	1.00
Primary education	0.64 (0.55–0.75)	0.52 (0.43–0.63)
Secondary education	0.52 (0.44–0.63)	0.18 (0.13–0.26)
Higher education	0.32 (0.17–0.60)	1.00
Place of residence		
Urban	1.00	1.00
Rural	1.18 (1.01–1.38)	1.24 (1.04–1.48)
Religion		
Muslim	0.01 (0.00–0.01)	0.02 (0.01–0.03)
Christian	0.001 (0.000–0.001)	0.007 (0.003–0.014)
Animist/other	1.00	1.00
Wealth index		
Poorest	0.76 (0.58–1.01)	0.76 (0.54–1.08)
Poorer	0.84 (0.65–1.09)	0.71 (0.50–0.99)
Middle	0.95 (0.75–1.19)	0.73 (0.53–1.01)
Richer	1.04 (0.84–1.29)	0.54 (0.38–0.76)
Richest	1.00	1.00
Ethnicity		
Wolof	1.00	1.00
Poular/Fulani	37.55 (30.2–46.8)	6.79 (4.93–9.35)
Serer	2.28 (1.62–3.22)	0.25 (0.09–0.70)
Mandingue	96.06 (69.0–133.7)	6.35 (4.37–9.23)
Diola	40.37 (29.1–56.0)	4.04 (2.49–6.54)
Soninke	71.96 (49.3–105.1)	6.22 (3.87–9.99)
Other	22.75 (17.55–29.5)	4.23 (2.92–6.13)
State of residence		
Dakar	4.85 (3.41–6.89)	0.51 (0.31–0.85)
Ziguinchor	23.54 (15.9–34.8)	2.69 (1.70–4.26)
Diourbel	1.00	1.00
Saint-Louis	12.32 (8.66–17.5)	1.76 (1.18–2.63)
Tambacounda	84.03 (56.9–124.2)	2.84 (1.93–4.18)
Kaolack	1.62 (1.09–2.40)	0.08 (0.03–0.22)
Thies	1.27 (0.82–1.97)	0.11 (0.04–0.31)
Louga	0.78 (0.52–1.18)	0.27 (0.16–0.45)
Fatick	3.47 (2.29–5.25)	0.21 (0.08–0.54)
Kolda	62.51 (42.7–91.4)	2.90 (1.97–4.26)
Matam	61.40 (42.0–89.8)	2.66 (1.82–3.90)
Kaffrine	4.16 (2.80–6.18)	0.20 (0.10–0.41)
Kedougou	162.6 (98.0–269.7)	0.87 (0.54–1.42)
Sedhiou	64.34 (42.9–96.5)	3.56 (2.38–5.33)

* Adjusted OR from standard logistic regression models for the sample of women respondents.

† Adjusted OR from standard logistic regression models for the sample of daughters.
